# Control of Molecular Ordering, Alignment, and Charge Transport in Solution-Processed Conjugated Polymer Thin Films

**DOI:** 10.3390/polym9060212

**Published:** 2017-06-08

**Authors:** Mincheol Chang, Gyun Taek Lim, Byoungnam Park, Elsa Reichmanis

**Affiliations:** 1 School of Polymer Science and Engineering, Chonnam National University, Gwangju 61186, Korea; gtlim@chonnam.ac.kr; 2 Department of Materials Science and Engineering, Hongik University, Seoul 121-791, Korea; metalpbn@gmail.com; 3 School of Chemical and Biomolecular Engineering, Georgia Institute of Technology, Atlanta, GA 30332, USA; 4 School of Chemistry and Biochemistry, Georgia Institute of Technology, Atlanta, GA 30332, USA; 5 School of Materials Science and Engineering, Georgia Institute of Technology, Atlanta, GA 30332, USA

**Keywords:** conjugated polymers, morphologies, charge transport, self-assembly, molecular ordering, polymer chain alignment, organic field-effect transistors

## Abstract

Morphology of conjugated polymers is a critical factor that significantly affects intrinsic charge transport characteristics and in turn performance of polymer-based devices. Morphological defects including misaligned crystalline grains and grain boundaries significantly impede efficient charge hopping between transport sites, resulting in degradation of device performance. Therefore, one important challenge is to control morphology of active polymer thin-films for achieving high performance flexible electronic devices. In the past decade, significant progress has been achieved in morphology control of conjugated polymer thin-films using solution-based processing techniques. This review focuses on recent advances in processing strategies that can tune the morphologies and thus impact charge transport properties of conjugated polymer thin films. Of the available processing strategies, polymer solution treatments and film deposition techniques will be mainly highlighted. The correlation between processing conditions, active layer morphologies, and device performance will be also be discussed.

## 1. Introduction

Conjugated polymers have attracted great interest for a variety of applications including thin-film field effect transistors (FETs) [1,2,3,4,5], light-emitting diodes (LEDs) [6,7,8,9,10], and photovoltaic cells (PVCs) [11,12,13,14,15,16], due to their low temperature, solution-based processability, which may enable low-cost, large area electronic device fabrication. However, conjugated polymer-based devices have been restricted in commercialization owing to their low device performance. In general, conjugated polymer films prepared via solution processing exhibit semi-crystalline structures in which many small crystals coexist with a largely disordered matrix [17,18,19,20]. Such structural disorder of the films is the obvious reason for the low device performance, which is unfavorable for efficient charge hopping between transport sites [5,17,18,19,20,21,22]. Accordingly, tremendous efforts have been made to enhance charge carrier mobility and in turn device performance via controlling morphology and alignment of the polymer thin-films in combination with synthetic chemistry approaches [1,2,23,24,25,26,27,28,29,30,31]. While molecular engineering approaches have successfully created a multitude of novel structured conjugated polymers using various synthetic routes and design principles [1,26,29], device performance has also been significantly improved by optimizing morphology and alignment of the polymer thin-films [2,23,24,25,27,28]. To date, various processing techniques have been developed and employed to control the morphology and alignment of conjugated polymer thin-films including polymer-dielectric interface modification [32,33,34,35], thermal annealing [36,37,38], solvent vapor annealing [30,39,40,41], film-deposition methods (e.g., spin-coating [42,43,44], drop-casting [36,45,46,47], dip-coating [48,49,50], and shear-coating [36,51,52,53,54,55,56]), and solution treatments (e.g., tuning solubility [56,57,58,59,60], solution aging [56,61], addition of nucleating agents [62,63,64], sonication [49,60,65,66,67], and UV irradiation [68,69,70]). Nevertheless, such techniques are still lacking in understanding on how to control processing parameters for achieving the desired performance of devices based on conjugated polymers.

While some papers have reviewed some solution treatments and film-deposition methods, they have not covered all the associated techniques [28,71,72,73]. Remarkably, multiple new alternative strategies have been reported in recent years [52,53,54,55,56,57,58,59,60,61,62,63,64,65,66,67,68,69,70]. Therefore, this article particularly reviews recent research efforts and progress in solution processing techniques such as polymer solution treatments and film deposition techniques used in research on conjugated polymer thin-film devices. In addition, the process-morphology-property relations of solution-processed conjugated polymer thin films will be discussed, which may offer a general guideline toward optimization of the morphology and alignment of conjugated polymers for maximizing device performance.

## 2. Solution Treatments

Typically, pre- and/or post-deposition processing techniques such as polymer-dielectric interface treatments [32,33,34,35], thermal annealing [36,37,38,74], and solvent vapor annealing [30,39,40,41] have been largely explored for improving the crystallinity, i.e., intra- and intermolecular interactions of conjugated polymers in the solid state, resulting in improved charge transport within the resultant films. For instance, Kang et al. demonstrated that the morphological and crystalline characteristics of pentacene thin films can be controlled by tuning the grain structure of a self-assembled monolayer of octadecyltrichlorosilane (OTS), on which the organic thin films were deposited [75]. Lee et al. significantly enhanced poly(3-hexylthiophene) (P3HT) field-effect mobility by annealing the polymer film at high temperature (~150 °C), which resulted in improved polymer crystallinity and contact between the polymer and device electrodes [76]. Fu et al. demonstrated enhanced molecular ordering of P3HT chains via exposure of the polymer film to o-dichlorobenzene vapor [77]. However, these approaches are not suitable for large-scale fabrication and high-throughput processing of polymer-based devices due to their complicated and tedious steps: self-assembled monolayer depositions, creation of oxygen- and moisture-free environments, high temperature treatments, and so on. Recently, a diverse set of solution treatment techniques, which can improve molecular ordering and charge transport characteristics of the resultant polymer films without the additional pre- and/or post-steps mentioned above, have been developed [55,56,57,58,66,67,68,69,70]. These techniques induce well-ordered nanoaggregates in solution, prior to or during film deposition. In this section, some representative solution treatment techniques reported in previous literature reports will be introduced and described.

### 2.1. Solvent Solubility Tuning

The physical interaction between solvent and polymer is one of the key parameters that govern the morphology of resultant polymer thin-films [28,40,56,57,58,67,68,69,70]. In general, approaches such as binary solvent systems [57,58,59], selection of marginal solvents [64,78,79], solution aging [56,60] and solution cooling [80,81] have been explored to adjust the interactions between solvents and polymers and thus morphology (i.e., degree of crystallinity, shape and size of crystallites, and phase separation) of solidified polymer films. Park et al. presented a solvent mixture system consisting of a small amount of higher boiling non-solvent acetonitrile (boiling point (bp) 81 °C) and a majority good solvent, chloroform (bp 61 °C), which led to improved two-dimensional molecular ordering of poly(3-hexylthiophene) (P3HT) chains and concomitant charge carrier mobility [57]. Surprisingly, a 20-fold enhancement (up to 1.5 × 10^−2^ cm^2^·V^−1^·s^−1^) in charge carrier mobility was achieved by addition of a very small amount of acetonitrile, ~3.3 vol %. It is believed that the less volatile poor solvent resides for a longer period of time within the evolving film, resulting in the formation of crystalline aggregates in resultant P3HT thin-films via an unfavorable interaction between the acetonitrile solvent and the poorly soluble P3HT.

**Figure 1:**
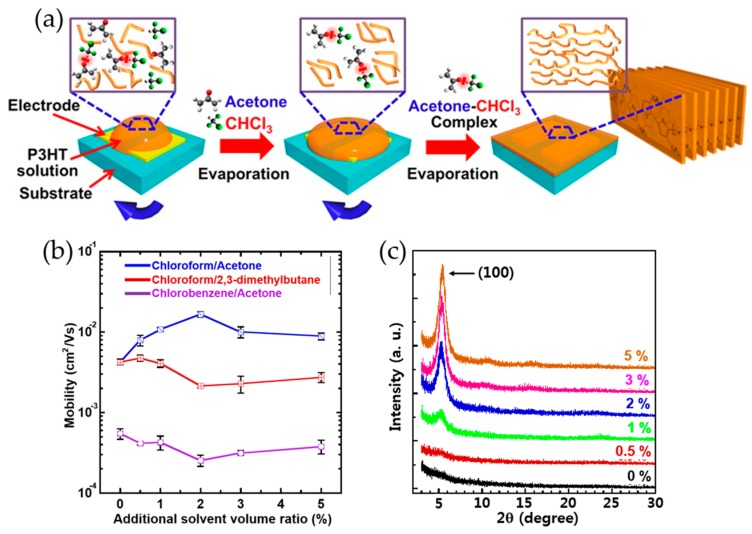
(**a**) Schematic illustration of evolution of molecular ordering of poly(3-hexylthiophene) (P3HT) chains during the deposition process; (**b**) average field-effect mobilities of P3HT films obtained via spin coating from chloroform/acetone, chlroform/2,3-dimethylbutane and chlorobenzene/acetone solvent blends as a function of poor solvent volume ratio; and (**c**) grazing incidence X-ray diffraction profiles of the P3HT films spin-coated from P3HT/chloroform solutions with different acetone concentrations. Reproduced with permission from [58]. Copyright 2013 American Chemical Society.

In contrast to the prevailing opinion mentioned above, Chang et al. demonstrated that incorporation of a lower boiling poor solvent that additionally interacts with the majority good solvent via hydrogen bonding could positively influence the molecular ordering and charge transport characteristics of conjugated polymers. In this study, acetone (bp 56 °C) and chloroform (bp 61 °C) were used as a volatile non-solvent and a majority good solvent for P3HT, respectively, given that the mixed solvent system is one of the most studied hydrogen-bonded complexes [82]. Figure 1a presents the schematic illustration of supramolecular assembly of P3HT chains during the deposition process. The characteristics of the binary solvent system vary from those of a good solvent to those of a poor solvent during solvent evaporation because the acetone-chloroform complexes that act as a poor solvent to P3HT evaporate more slowly than respective single component solvents. As a result, the supramolecular assembly of P3HT chains is considerably improved. Consequently, the macroscopic charge carrier mobility was dramatically enhanced, reaching a maximum (1.7 × 10^−2^ cm^2^·V^−1^·s^−1^) at 2.0 vol % of acetone (Figure 1b). However, a further increase in acetone content led to a decrease in mobility, likely because the number of interfaces between crystalline domains may begin to increase while the crystallinity of the polymer thin-films continues to increase with an increase of acetone concentration (Figure 1c).

He et al. demonstrated that crystalline nanostructures such as nanowires and nanorings of the all-conjugated diblock copolymer poly(3-butylthiophene)-*b*-poly(3-hexylthiophene) (P3BHT) can be produced by manipulating the anisole/chloroform ratio [59]. Chloroform is a good solvent while anisole is a marginal solvent for both blocks. The marginal solvent is defined as a solvent that can act as a good solvent at higher temperature, but is a poor solvent at ambient temperature. The self-assemblies of P3BHT were induced during cooling of the solution by solvophobic interactions between the polymer blocks and the marginal solvent anisole to minimize the unfavorable contacts between the solute and solvent components.

**Figure 2:**
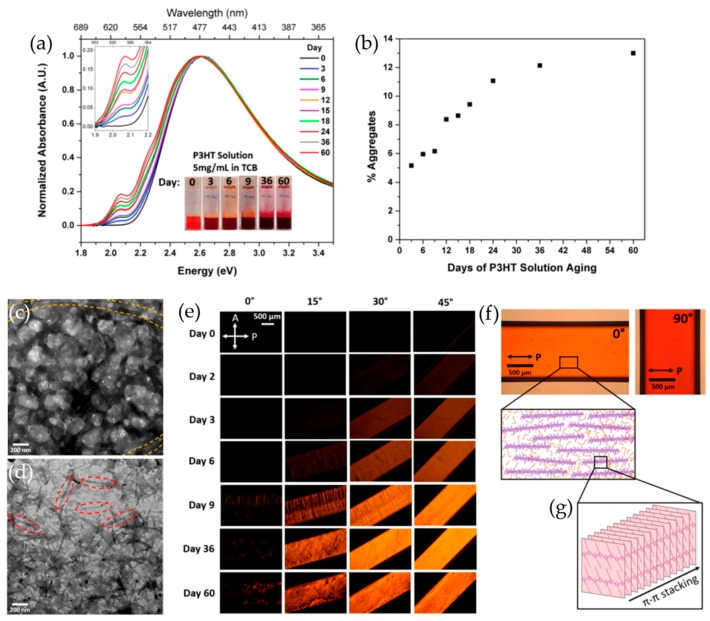
(**a**) Solution UV-vis spectra; and (**b**) percent aggregates of P3HT/1,2,4-trichlorobenzene (TCB) solution as a function of time; Cryo-transmission electron microscopy (TEM) images of P3HT solutions in TCB aged for: (**c**) 0 and (**d**) 31 days. (**e**) Polarized optical microscopy (POM) images of capillaries filled with P3HT/TCB solutions aged for a range of times, rotated in increments of 15° between crossed polarizers. (**f**) Change in absorption of a capillary filled with a P3HT/TCB solution aged for 36 days as a function of capillary orientation with respect to polarizer. (**g**) Schematic representation of P3HT nanofibers aligned along the capillary long axis. Reproduced with permission from [60]. Copyright 2015 American Chemical Society.

Recently, Kleinhenz, et al. presented the time-dependent self-assembly process of P3HT in 1,2,4-trichlorobenzene (TCB) solutions [60]. Figure 2a shows the electronic absorption spectra obtained from a P3HT/TCB solution aged at different times. As the solution aging time increased, the solution became dark, and the vibronic peak at ~599 nm began to develop, indicating an increased portion of P3HT aggregates formed via improved co-facial π–π stacking [58,66]. Consistently, the amount of aggregates increased up to ~13% with an increase in aging time from 0 to 60 days (Figure 2b). The P3HT aggregates in solution were revealed to be one-dimensional nanostructures by cryo-TEM image analysis as shown in Figure 2c,d. The freshly prepared solution was observed to be amorphous, whereas nanofibrillar structures appeared in the aged solution. They further showed that the aggregates can be readily oriented parallel to the capillary long axis, which is evidenced by polarized optical microscopy as seen in Figure 2e–g. A capillary filled with fresh P3HT/TCB solution (Day 0) appeared dark and therefore isotropic while capillaries filled with aged solutions exhibited increasingly bright birefringent textures as the aging time increased up to 60 days. Moreover, the alignment of aggregates was proven to be preserved into the solid state via a drawing of the aged solutions onto substrates, implicating the improved charge transport in electronic devices based on conjugated polymers.

Solvent solubility relative to a conjugated polymer was also modulated by varying the temperature of the polymer solution [80,81]. For example, Lee et al. prepared crystalline P3HT nanofibrils using a cycle of cooling and heating in P3HT solutions [80]. The m-xylene solutions of the polymers were cooled to −20 °C at a rate of −8 °C/min and reheated to 25 °C at a rate of 1.8 °C/min, respectively. Solubility of solutes generally decreases with decreased temperature. Hence, P3HT nanocrystals were formed and then grew into fibrillary structures as temperature was decreased. Meanwhile, the as-prepared P3HT nanofibrils were not dissolved although temperature was increased up to 25 °C, owing to strong π−stacking interactions. Growth of nanofibrils during the cooling process was monitored by UV-vis spectroscopy; the absorbance at 560 and 605 nm ascribed to π–π stacking began to appear at 10 °C and became prominent as the solution temperature was further decreased to −20 °C.

### 2.2. Nucleation-Inducing Agent

The formation of polymer crystallites can be also triggered and controlled by heterogeneous additives in addition to solvent solubility tuning [61,62,63]. Such additives, so-called nucleating agents, provide a heterogeneous surface for the polymer melts or solutions, which facilitates a thermodynamically favorable crystallization of the polymers. As a result, the temperature, at which crystallization of the polymers begins, becomes elevated. For example, Liu et al. demonstrated the formation of 2D crystalline P3HT/carbon nanotube (CNTs) supramolecular structures using a controlled polymer crystallization method [61]. In situ UV-vis spectroscopy revealed that CNTs facilitate P3HT crystallization and P3HT nanowire formation is governed by first-order kinetics. Crystalline P3HT nanowires were grown perpendicular from the CNT surfaces, which is attributed to the epitaxial interaction between the P3HT backbone and the surface of the CNTs.

**Figure 3:**
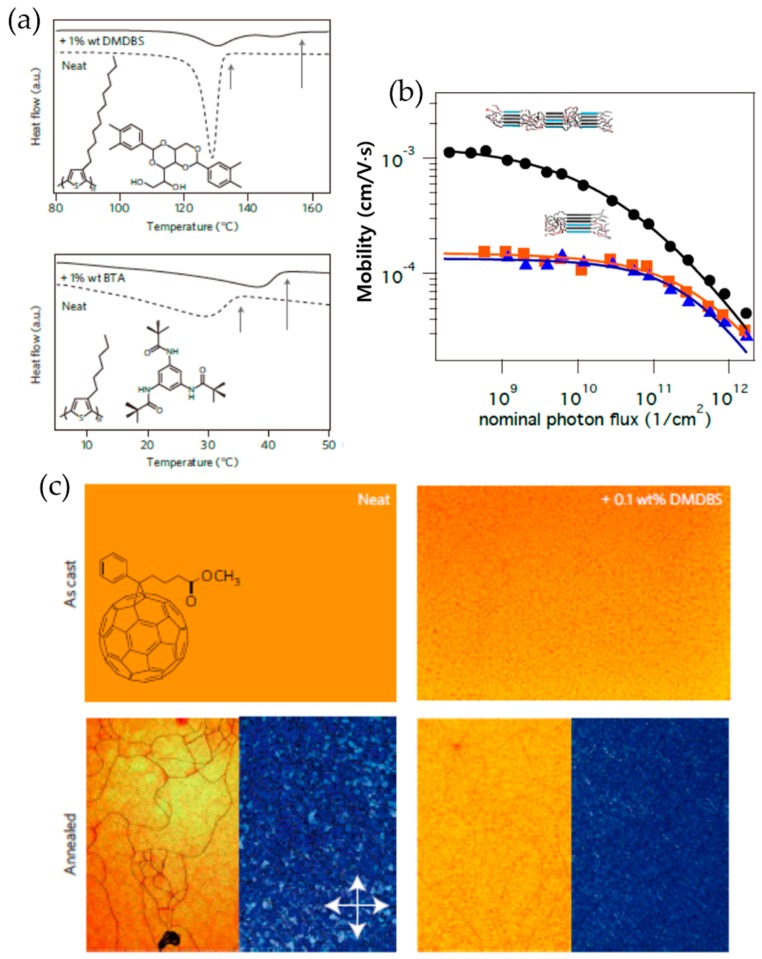
(**a**) Differential scanning calorimetry (DSC) cooling thermograms of poly(3-dodecylthiophene) (P3DDT) (top) and P3HT (bottom) on the addition of nucleation agents, 1,3:2,4-bis(3,4-dimethylbenzylidene)sorbitol (DMDBS) and tris-*tert*-butyl-1,3,5-benzenetrisamide (BTA). (**b**) Time-resolved microwave conductivity measurements of yield-mobility product of P3DDT, neat (orange squares) and comprising DMDBS (black circles) or BTA (blue triangles). (**c**) Transmission optical micrographs of neat [6,6]-phenyl-C_61_-butyric acid methyl ester (PCBM) drop cast at room temperature from chlorobenzene (left) and with 0.1 wt % DMDBS (right) before and after thermal annealing at 180 °C for 30 min in a nitrogen environment. Bottom right subpanels are cross-polarized transmission micrographs for the corresponding annealed films. Reproduced with permission from [62]. Copyright 2013 Nature Publishing Group.

Recently, Treat et al. induced nucleation of conjugated polymers such as poly(3-dodecylthiophene) (P3DDT), P3HT, and [6,6]-phenyl-C_61_-butyric acid methyl ester (PCBM) using two inert additives (i.e., 1,3:2,4-bis(3,4-dimethylbenzylidene)sorbitol (DMDBS) and tris-*tert*-butyl-1,3,5-benzenetrisamide (BTA)) that are typically designed for melt solidification of isotactic polypropylene [62]. The additives were initially mixed at the molecular level with the molten polymer or solution, and facilitated heterogeneous nucleation of the host materials on their well dispersed, nanoscopic surfaces upon cooling and solvent removal. Figure 3a depicts the differential-scanning-calorimetry (DSC) cooling thermograms for P3DDT and P3HT upon the addition of DMDBS and TBA, respectively. The crystallization temperature *T*_c_ of P3DDT increased by as much as 24 °C, from 134 to 158 °C, on the addition of only 1 wt %, which was recorded during cooling of the polymer melt. A similar behavior was found when a small amount of nucleation agent, BTA was added to a P3HT melt. An increased *T*_c_ (from 36 to 43 °C) was observed from the P3HT melt in the presence of 1 wt % BTA, which indicates an increased rate of crystallite nucleation of the polymer. Interestingly, the charge carrier mobility of the polymers was significantly affected by the addition of the nucleation agents while no discernable change in the degree of crystallinity of the resultant polymers was observed. In particular, the addition of DMDBS into P3DDT led to an increased charge carrier mobility by up to 50%, which is probably due to the creation of a larger interfacial area between crystalline and amorphous domains (Figure 3b). However, BTA provided no perceptible change in nucleation surface area and concomitantly, an indistinguishable change in the mobility, which is attributed to the lower solubility of the nucleation agent in P3DDT. In addition, a minute amount of DMDBS profoundly affected the solidification of PCBM by forming high-surface-area structures that induce heterogeneous nucleation without adversely affecting charge transport properties of the polymer. As observed in Figure 3c, the annealed PCBM domain size was decreased to less than 1 μm upon the addition of 0.1 wt % DMDBS while the annealed neat films exhibited randomly oriented, crystalline domains with a size of ~20 μm. These results indicate that the dimensions of polymer crystallites can be simply tuned by the addition of the nucleation agents.

More recently, Rosu et al. reported that aqueous dispersions of cerato ulminm (CU), which is a class II hydrophobin protein, can facilitate enhanced alignment and organization of P3HT chains in solution and in turn, highly crystalline P3HT structures in the solidified films [63]. In a typical aqueous suspension process, a solution of P3HT in TCB was introduced into an aqueous dispersion of the hydrophobin, CU. Upon mild agitation, the polymer solution gradually diffused into and then was confined within CU membrane-stabilized microstructures due to the hydrophobic character of the polymer. In those confinements, the molecular alignment of polymer chains was improved by a transition from isotropic to liquid crystalline fluid to polycrystalline states. Despite significantly aligned and organized structures of P3HT, the charge-carrier mobility of resultant P3HT/CU films was measured to be much lower than expected because the presence of CU led to poor film uniformity on the active channel between source and drain electrodes.

### 2.3. Ultrasonic Treatment

**Figure 4:**
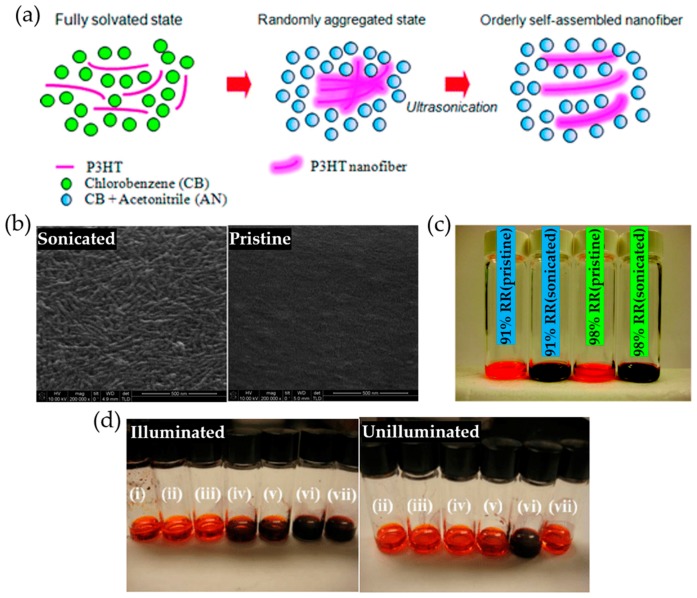
(**a**) Schematic illustration of P3HT aggregation in a solvent system containing a dipolar solvent. (**b**) Scanning electron microscopy (SEM) images of P3HT films prepared from a respective ultrasonicated solution (10 mg of P3HT in 1 mL of chlorobenzene (CB) (95 vol %)/acetonitrile (5 vol %) cosolvent) and pristine solution (10 mg of P3HT in 1 mL of CB). All images were taken using 98% regioregular (RR) P3HT, and films were not thermally treated after spin-casting. Scale bar is 500 nm. (**c**) Aggregation behavior of P3HT in CB containing 5 vol % of acetonitrile upon ultrasonication treatment: 91% RR P3HT without ultrasonication, 91% RR P3HT with ultrasonication, 98% RR P3HT without ultrasonication, and 98% RR P3HT with ultrasonication. The image was taken 2 h after applying ultrasonication for 2 min. (**d**) Illumination effect on the aggregation behavior of P3HT in various solvent systems with different polarities and solubilities: (i) CB; (ii) CB/hexane; (iii) CB/1,2-dichloromethane; (iv) CB/chloroform; (v) CB/methanol; (vi) CB/acetonitrile; and (vii) CB/dimethylformamide. All solutions were illuminated under room light for 2 h without ultrasonication. Reproduced with permission from [65]. Copyright 2010 American Chemical Society.

Ultrasonication has been generally utilized in many applications and processes such as homogenization [83], disintergration [84], sonochemistry [85], degassing [86], and cleaning [87]. In addition, it has been used to nucleate and grow molecular crystals [88,89,90]. Meanwhile, it has seldom been employed in polymer processing to form polymer crystallites, given that disaggregation behavior of polymers under ultrasonication is commonly reported [91,92,93]. Recently, ultrasonication has proven to be useful in initiating and promoting the nucleation and crystallization of conjugated polymers [49,64,65,66,67]. As hypothesized by Zhao et al., ultrasonic irradiation could promote disorder–order transformation within the individual polymer chains in solution, giving rise to co-facial stacking between the polymer chains [64]. In other words, the acoustic cavitation process involved in ultrasonication facilitates chain disentanglement, followed by a shear induced change of the polymer chain conformation, resulting in well-ordered polymer aggregates formed through π–π interactions. Kim et al. presented a sonication-assisted self-assembly method that led to the formation of well-ordered P3HT nanowires [65]. The P3HT self-assembly was facilitated by adding a polar poor solvent, acetonitrile into a nonpolar good solvent under ultrasonication treatment. On the other hand, the addition of nonpolar poor solvent, hexane led to no aggregation of P3HT. Accordingly, it was asserted that the self-assembly behavior of the polymer greatly depends on the solvent polarity rather than the solubility power under a sonication process. They suggested a possible mechanism for the self-assembly of P3HT induced by ultrasonication (Figure 4a). According to their hypothesis, as the solvent polarity increases, the interaction between P3HT and the solvent decreases while the interaction between the polymer chains become more favorable. Consecutively, P3HT chains slowly aggregate into low order crystalline structures, in which the polymer chains are randomly entangled. Once ultrasonic irradiation is applied to the aggregates in solution, the single polymer chains are separated from the aggregates, and then aggregate again due to the unfavorable interaction between the polymer and the polar solvent. After multiple cycles of such aggregation and disaggregation processes, highly ordered, namely crystalline, P3HT nanofibrillar aggregates are finally obtained as shown in Figure 4b. Additionally, they found that several factors including regioregularity of P3HT and illumination conditions, can affect the self-assembly of P3HT. The higher regioregularity of P3HT led to a larger concentration of the polymer aggregates after ultrasonic treatment (Figure 4c). A dark purple color was observed from the 98% regioregular (RR) P3HT solution while a lighter purple color appeared from the 91% RR P3HT solution. The aggregation of the polymer appeared preferable when illuminated under ambient light vs. a dark condition (Figure 4d). Similarly, Aiyar et al. demonstrated that ultrasonic irradiation can lead to the formation of well-ordered nanofibrillar aggregates of P3HT in solution and further, that the amount of aggregates can be controlled by sonication time [66]. Surprisingly, the P3HT films obtained from an ultrasonicated solution exhibited an order of magnitude enhancement (from ~1.6 × 10^−4^ to 1.4 × 10^−2^ cm^2^·V^−1^·s^−1^) in mobility, compared to pristine P3HT films. No discernable polymer degradation was observed after ultrasonic irradiation because the exposure dose (0.4 W cm^−2^, 40 kHz or less than 10 min) used in their experiments was far lower than that (6.8 W cm^−^^2^, 960 kHz for 2 h) required to cause polymer degradation.

**Figure 5:**
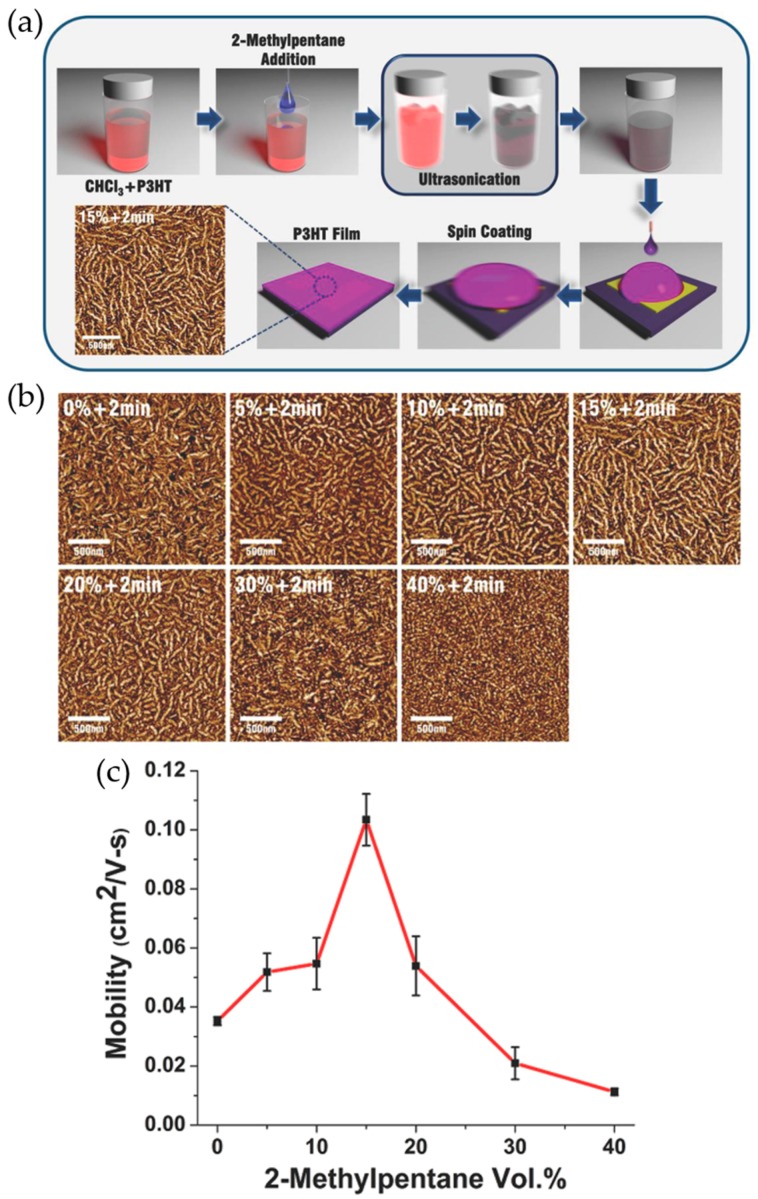
(**a**) Schematic illustration of P3HT nanorod preparation through a combined ultrasonication/nonsolvent addition approach; (**b**) tapping mode atomic force microscopy (AFM) phase images of P3HT films obtained by spin coating chloroform-P3HT solutions with varying proportions of nonsolvent (2-methylpentane) followed by ultrasonication for 2 min; and (**c**) average field-effect mobilities of P3HT films spin coated from chloroform-P3HT solutions with corresponding proportion of 2-methylpentane followed by 2 min of ultrasonication. Reproduced with permission from [67]. Copyright 2015 WILEY-VCH Verlag GmbH & Co. KGaA, Weinheim.

Recently, synergistic combination of ultrasonic irradiation and nonsolvent addition was reported by Choi et al., which led to controlled P3HT self-assembly into highly ordered rod-like nanostructures with varied length through a simple 2-step crystallization process (i.e., nucleation followed by growth) [67]. The synergistic combination of nonsolvent addition followed by ultrasonication significantly improved charge transport of the resultant films, compared to each method used alone. Figure 5a depicts the controlled P3HT assembly into nanorod-like crystalline structures via a combined ultrasonication/nonsolvent addition strategy. Firstly, a requisite amount of the nonsolvent, 2-methylpentane is introduced into a P3HT/chloroform solution in order to modulate the solution solubility, by which the crystal nucleation or growth is critically affected. Subsequently, ultrasonic irradiation is applied to the resultant P3HT solution to nucleate and grow P3HT nanocrystallites. Finally, P3HT films are obtained by spin coating the corresponding solutions. The crystallization processes (i.e., nucleation and growth from solution) strongly depend on the degree of supersaturation. When a solution is moderately supersaturated (metastable state), crystal growth that is initiated from existing nucleation sites is preferred to the formation of new nucleation sites. Meanwhile, as the solution becomes further supersaturated (unstable state), the nucleation of excessive solute is dominantly triggered over crystal growth. It was revealed that the addition of 2-methylpentane profoundly impacts the crystallization processes of P3HT in solution, resulting in morphological change in the solidified films. As shown in Figure 5b, the P3HT nanorods increased in length as the 2-methylpentane content increased up to 15%, indicative of dominant crystal growth in a metastable solution. Whereas, the length of the structures remarkably decreased with further increased nonsolvent content, which indicates that the solutions became unstable and thus subject to spontaneous nucleation. The charge carrier mobility of resultant films was dramatically improved with increased P3HT nanorod length, owing to a decreased number of grain boundaries impeding efficient charge hopping between transport sites (Figure 5c).

### 2.4. UV Irradiation

**Figure 6:**
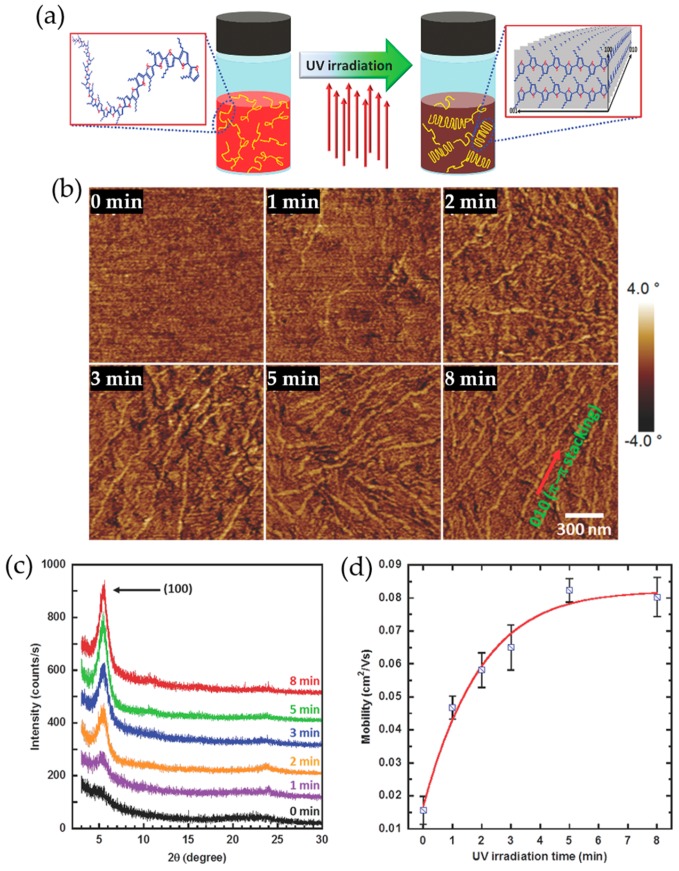
(**a**) Suggested mechanism for the UV irradiation induced anisotropic molecular ordering of P3HT chains; (**b**) tapping mode AFM phase images of P3HT films obtained by spin coating chloroform-P3HT solutions irradiated by UV for 0, 1, 2, 3, 5, and 8 min; (**c**) grazing incidence X-ray diffraction profiles; and (**d**) average field-effect mobilities of corresponding P3HT films. Reproduced with permission from [68]. Copyright 2014 WILEY-VCH Verlag GmbH & Co. KGaA, Weinheim.

Another effective approach to morphology control of conjugated polymer films is UV irradiation of the precursor solutions. Accidently, Chang et al. found that low intensity, limited duration UV irradiation can enhance anisotropic supramolecular assembly of P3HT chains in solution with no discernable degradation of the polymer chains, resulting in a significant enhancement in charge carrier mobility of resultant films (Figure 6) [68]. It was believed that a conformational change of the polymer chains from aromatic- to quinoid-like occurs upon photoexcitation by UV irradiation, which facilitates supramolecular self-assembly of P3HT into highly crystalline nanofibrillar aggregates through favorable π–π interactions between the polymer chains; in the excited state, the polymer chains are coplanarized due to enhanced π-orbital overlap between individual thiophene rings (Figure 6a). The nanofibrillar aggregates formed in solution survived a spin-coating process, and in turn evidently provided the resultant films with improved crystallinity and charge transport characteristics (Figure 6b–d). The crystalline nanofibrillar aggregates appeared to become increased in both length and concentration with increased UV irradiation time, contributing to dramatic enhancement of charge carrier mobilities of the resultant films, presumably due to a reduction in the number of grain boundaries.

Further, they demonstrated that UV irradiation of blended polymer (polymer semiconductor/insulating polymer) solutions can be a facile and effective alternative to achieve high-performance, low-cost and environmentally stable semiconductor/insulator polymer blends for device applications [69]. P3HT nanofibrillar structures with a high degree of molecular ordering were formed in the presence of an insulating polymer, polystyrene (PS) in solutions through low intensity, limited duration UV irradiation. As a consequence, the P3HT islands separately enclosed in PS rich regions were effectively connected by the P3HT nanofibrillar aggregates within the blend films, resulting in excellent charge transport characteristics, even at a P3HT content as low as 5 wt %. The environmental stability of the blend-based OFETs in ambient condition was also significantly improved by the PS layers that protect P3HT from exposure to damaging influences such as moisture or oxygen.

Both crystallinity (i.e., molecular ordering) and grain boundaries in conjugated polymer thin films are dominant key factors that impact the charge transport characteristics of the polymer films. In order to maximize charge transport, the enhancement of molecular ordering between polymer chains is imperative while the suppression of grain boundary formation is desirable. As aforementioned, ultrasonication of polymer solutions is an effective technique to nucleate the polymer crystallites. However, it restricts the growth of crystalline nanofibrillar structures to less than ~200 nm in length, due to strong ultrasound agitation, producing a number of grain boundaries that impede efficient charge transport between crystalline grains within resultant films. In contrast, UV exposure to polymer solutions tends to provide for fibrillar structures longer than ~1 μm while it affords films possessing a relatively lower degree of crystallinity.

**Figure 7:**
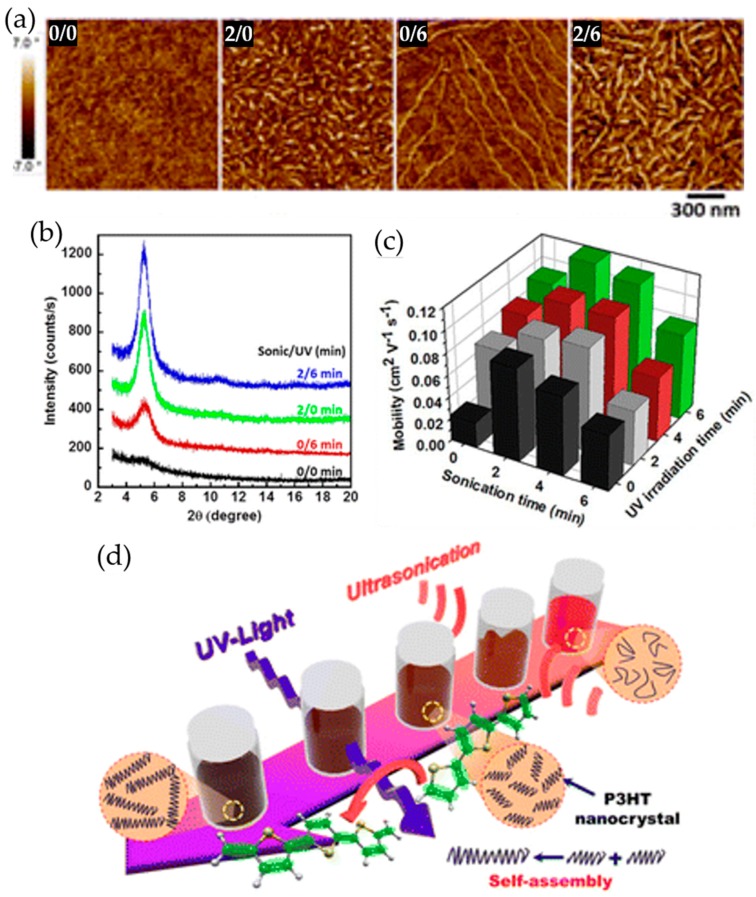
(**a**) Tapping mode AFM phase images of P3HT films spin coated from the polymer solutions treated by ultrasonication and subsequent UV irradiation for 0 and 0 min, 2 and 0 min, 0 and 6 min, and 2 and 6 min, respectively; (**b**) grazing incidence X-ray diffraction profiles of corresponding films; (**c**) average field-effect mobilities of P3HT films obtained by spin coating the polymer solutions exposed to ultrasonication and/or UV irradiation for various times; and (**d**) the Suggested mechanism describing the anisotropic assembly of P3HT nanocrystallites formed by ultrasonication into longer nanofibrillar structures via subsequent UV irradiation of the solution. Reproduced with permission from [70]. Copyright 2014 American Chemical Society.

In recent years, Chang et al. reported a synergistic combination of ultrasonication followed by UV irradiation of polymer solutions, which led to a remarkable improvement in mobility of corresponding films with commensurately desirable morphology [70]. They exposed P3HT solutions to a sequential ultrasonication and UV irradiation process for times ranging from 0 to 6 min, respectively. Surprisingly, the short nanofibrillar structures obtained via ultrasonic treatment for 2 min grew into longer units by subsequent UV irradiation for 6 min (Figure 7a). The sequential treatment also afforded a relatively higher degree of crystallinity of resultant films (Figure 7b). Consistent with atomic force microscopy (AFM) and X-ray diffraction (XRD) data, the charge transport characteristics of resultant films were further improved by the sequential ultrasonication and UV irradiation process, compared to treatment via either method alone (Figure 7c). In particular, the charge carrier mobility of OFET devices based on P3HT films, which were prepared by spin coating the precursor solutions treated through 2 min ultrasonication followed by 6 min UV irradiation, was enhanced by approximately 6-fold, in comparison to that of pristine P3HT film based OFET devices. They believed that the conformation of polymer chains present at the ends of the small crystallites that are formed by ultrasonication changes from random coil (aromatic) to linear or extended (quinoid) when the polymer chains are photoexcited upon UV irradiation; the small crystallites self-assemble into longer fibrillar structures through π–π interactions between the photoexcited polymer chains existing at the ends of the neighboring crystallites (Figure 7d).

## 3. Film Deposition Techniques

The development of film deposition processes plays a critical role in the development of high performance organic electronic devices essential for a large variety of commercial applications; as well as the molecular ordering of polymer thin films, their alignment would be profoundly influenced by the film deposition processes, both of which strongly affect the polymer film based device performance [28,31,51]. Thus, a great deal of effort has been made to develop alternative film deposition techniques which can afford oriented thin films with desired charge transport characteristics [53,54,55,56]. Recently, articles have appeared that review thin film deposition methodologies such as conventional techniques (e.g., spin-coating and drop-casting), printing methods (e.g., ink-jet printing, aerosol-jet and spray printing, screen-printing, stamping, etc.), and meniscus-guided coatings including dip-coating, blading, template-guided solution-shearing, and so on [28,71]. In particular, template-guided solution-shear coating was revealed to be a more effective way to facilitate alignment in polymer thin films. Accordingly, many researchers have continuously made significant efforts to develop alternative solution shear coating techniques that are scalable to industrial manufacturing processes. In addition to the template-guided solution-shear coatings, alternative deposition methods using external forces such as capillary, centrifugal, and mechanical force (e.g., rubbing and compressing) have recently proved effective for improving the alignment of polymer thin films [94,95,96,97,98,99,100,101,102,103,104,105]. Therefore, we will review template-guided solution-shear coating methods and alternative deposition approaches using an external force with respect to recent research effort and progress that has been made in those deposition techniques.

### 3.1. Template-Guided Solution-Shear Coating

In contrast to typical blade coating techniques, a blade fully covers a small-molecule or polymer solution droplet on a substrate so that solvent evaporation occurs only at the edges of the droplet. The blade moves relative to the substrate in a given direction, allowing for solvent evaporation followed by the deposition of aligned resultant films. Since Bao’s group reported a template-guided solution-shearing method as an effective tool for the deposition of highly aligned small-molecule single crystals with a high charge carrier mobility [51], this method has been extensively applied to conjugated polymer systems.

**Figure 8:**
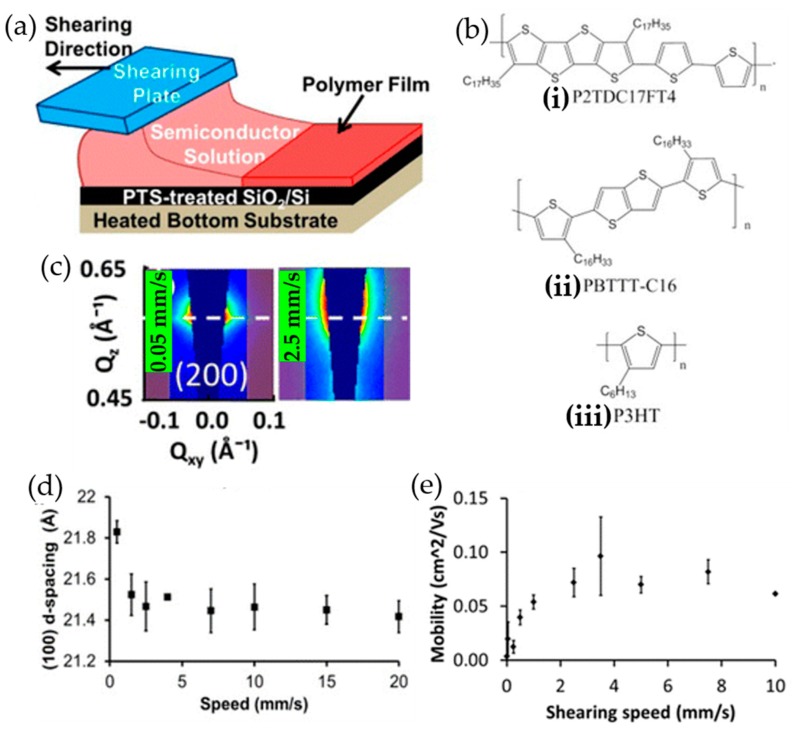
(**a**) Schematic illustration of solution shearing. (**b**) Chemical structures of polymers used in solution shearing: (i) P2TDC17FT4; (ii) PBTTT-C16; (iii) P3HTT. (**c**) The (200) Bragg peak position of a P2TDC17FT4 thin film solution sheared at 0.05 and 2.5 mm/s. The white dashed line indicates the (200) Bragg peak position of a dropcast sample. (**d**) Lamellar spacing of P2TDC17FT4 thin films as a function of shearing speed. (**e**) Average mobilities of the solution sheared P2TDC17FT4 TFTs as a function of shearing speeds. The error bars show the standard deviation. Reproduced with permission from [53]. Copyright 2015 American Chemical Society.

For instance, Khim et al. reported a simple wire-bar-coating process that can lead to a highly crystalline conjugated polymer layer on a substrate [52]. In a typical film deposition process, a small amount of polymer solution comprising alkyl-substituted thienylenevinylene and phthalimide-based donor-acceptor copolymer (PTVPhl-Eh) or diketopyrrolopyrrole thieno [3,2-b] thiophene (DPPT-TT) was dropped onto one edge of the patterned substrate. Then, a bar wrapped with wire coils was lowered onto the solution droplet and horizontally moved at a given speed over the substrate, resulting in a uniformly deposited polymer film. The bar-coated conjugated polymer films exhibited higher crystallinity and commensurately enhanced charge carrier mobility (~0.24 for PTVPhl-Eh and 1.64 cm^2^·V^−1^·s^−1^ for DPPT-TT) as compared to spin-coated samples (~0.13 for PTVPhl-Eh and 0.72 cm^2^·V^−1^·s^−1^ for DPPT-TT). Giri et al. investigated the effect of a template-guided solution shearing method on the resultant film morphology of three conjugated polymers, poly(2,5-bis(thiophene-2-yl)-(3,7-dihepta-decanyl tetrathienoacene) (P2TDC17FT4), poly(2,5-bis(3-hexadecylthiophene-2-yl)thieno[3,2-b]thiophene) (PBTTT-C16), and P3HT (Figure 8a,b) [53]. The packing structures (i.e., lamellar spacing and conjugated polymer backbone tilt angle) of the interdigitated polymers such as P2TDC17FT4 and PBTTT-C16 were influenced by the solution shearing while no change in the molecular packing was observed from the solution-sheared noninterdigitated polymer (i.e., RR P3HT) films. In particular, the packing structures of P2TDC17FT4 films were more sensitively changed by varying the solution shearing speed: as the shearing speed was increased from 0.05 to 2.5 mm/s, the lamellar spacing was decreased from 21.9 ± 0.05 up to 21.3 ± 0.14 Å (Figure 8c,d) and the π-system average tilt angle was also decreased from 82.5 ± 2.1 to 67.6 ± 1.5°. Consequently, the mobility of P2TDC17FT4 was enhanced by a factor of 5 (i.e., from ~0.02 to ~0.1 cm^2^·V^−1^·s^−1^) as shown in Figure 8e. Shin et al. presented a template-guided solution shearing technique using a well-defined micro-patterned polydimethylsiloxane (PDMS) template [54]. Micropatterned polymer prisms composed of diketopyrrolopyrrole, thiophene, and thienothiophene units were deposited by guidance of the PDMS template with uniaxially aligned microgrooves. The anisotropic orientation of the polymer chains along the shear direction resulted in significant enhancement (from ~3.1 to ~7.3 cm^2^·V^−1^·s^−1^) in the charge carrier mobility of resultant polymer films.

**Figure 9:**
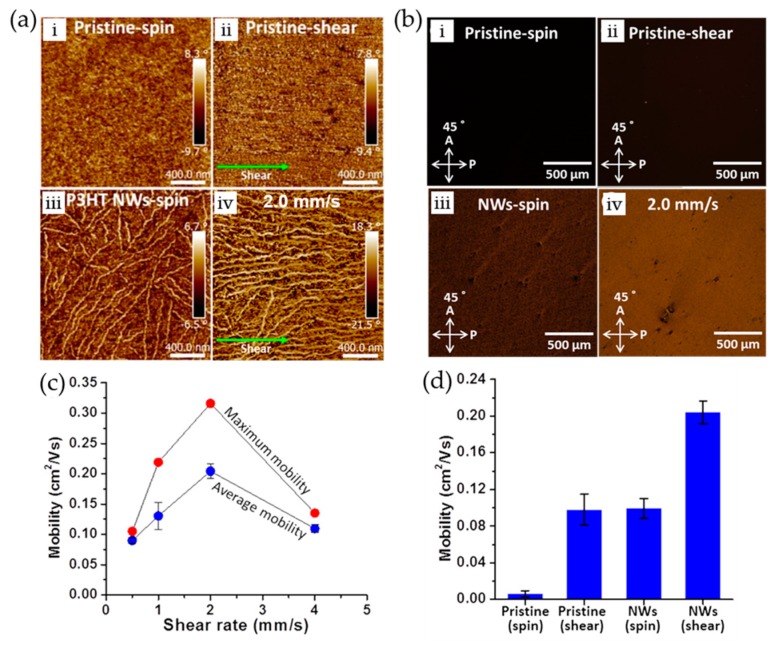
(**a**) AFM and (**b**) POM images of pristine P3HT thin films: (i) spin-coated; and (ii) shear-coated at 2.0 mm/s from a pristine solution; and P3HT NW films: (iii) spin-coated; and (iv) shear-coated at 2.0 mm/s from a P3HT solution containing P3HT NWs. (**c**) Average and maximum field-effect mobilities of P3HT NW films shear-coated as a function of the shearing speed. (**d**) Mobility comparison of P3HT pristine films spin-coated and shear-coated at 2.0 mm/s and P3HT NW films spin-coated and shear-coated at 2.0 mm/s. Reproduced with permission from [55]. Copyright 2016 American Chemical Society.

For conjugated polymers, solution shearing techniques still tend to afford locally ordered crystalline domains due to their multiple degrees of conformational freedom while it has successively led to aligned single-crystalline morphology for small molecules [28,51,52,53,54,55,56]. Hence, the mobility enhancement is observed to be relatively lower from the sheared polymer films vs. sheared small molecule films [28,53,54]. In order to reduce the concentration of grain boundaries formed within sheared polymer films, Chang et al. aligned micrometer-long P3HT crystalline nanowires (NWs) within resultant films via a solution shear coating of P3HT polymer solutions containing the NWs [55,56]. Compared to spin-coated pristine, sheared pristine, and spin-coated P3HT NW films, the sheared P3HT NW films exhibited more enhanced orientation and concomitantly, brighter birefringent textures as shown in Figure 9a,b. Additionally, intra- and intermolecular ordering and co-planarization of the polymer chains were enhanced by the solution shearing. As a result, the charge carrier mobility of P3HT NW films sheared under a speed of 2.0 mm/s was increased up to ~0.32 cm^2^·V^−1^·s^−1^, which is approximately 53-fold greater than the mobility (~0.6 × 10^-^^2^ cm^2^·V^−1^·s^−1^) of spin-coated pristine films (Figure 9c,d). Further, this approach was confirmed to be applicable to other conjugated polymers by demonstrating significantly improved alignment and charge carrier mobility of sheared poly(3-butylthiophene) (P3BT) NW films.

### 3.2. Capillary Force-Assisted Film Deposition

**Figure 10:**
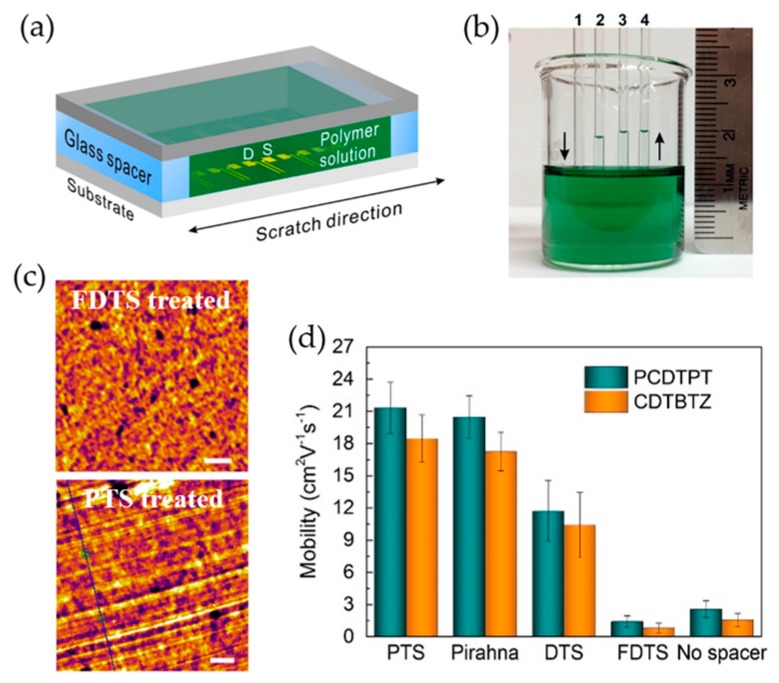
(**a**) Schematic illustration of the sandwich tunnel system consisting of two silicon substrates with a pair of glass spacers inserted at two ends. D and S denote the bottom-contact drain and source electrode, respectively. (**b**) Capillary height tests on polymer solution prepared in chlorobenzene using glass capillary tubes (inner diameter: ca. 1.2 mm) with various surface treatments: (1) perfluorodecyltrichlorosilane (FDTS); (2) n-decyltrichlorosilane (DTS); (3) piranha solution; (4) 6-phenylhexyltrichlorosilane (PTS); and (**c**) AFM images of the bottom surfaces of two deposited films approaching FDTS-treated spacer (top) and PTS-treated spacer (bottom). Scale bars represent 200 nm. (**d**) Comparison of the average saturation mobility of the devices prepared from poly[4-(4,4-dihexadecyl-4*H*-cyclopenta[1,2-b:5,4-b′]dithiophen-2-yl)-*alt*-[1,2,5]-thiadiazolo[3,4-c]pyridine] (PCDTPT) and poly[2,6-(4,4-*bis*-alkyl-4*H*-cyclopenta-[2,1-b;3,4-b0]-dithiophene)-*alt*-4,7-(2,1,3-enzothiadiazole)] (CDTBTZ) with various surface treatments over the pair of spacers. Reproduced with permission from [95]. Copyright 2014 American Chemical Society.

Capillary force that is involved with the interactions between liquid molecules and surrounding solid surfaces tends to drive a solution to flow and becomes more prominent due to the large surface to volume ratio when a solution is placed inside a narrower channel. Recently, several alternative strategies using capillary action have been reported [94,95], which can afford highly aligned conjugated polymer thin films. For instance, Higashi et al. demonstrated a film deposition technique that can facilitate self-assembly of polymer chains and unidirectional alignment of the polymer films with the assistance of capillary action [88]. A drop of polymer solution was introduced into one end of the gap between a glass fiber and substrate. Spontaneously, it flowed along the longitudinal direction in the gap by capillary action. Processed by this approach, thiophene-based polymer films such as PBTTT and poly(2,5-bis(3-alkylthiophene-2-yl)thieno[2,3-b]thiophene) (PBTCT) exhibited unique optical and electrical anisotropies. More recently, Luo et al. presented a general strategy for polymer chain self-assembly and unidirectional alignment on nanogrooved substrates using capillary action in a sandwich casting system [95]. A polymer solution was trapped due to surface tension in the tunnel composed of two Si/SiO_2_ substrates separated by two glass spacers on both short sides as shown in Figure 10a. Subsequently, self-assembly of polymer chains was effectively induced along the uniaxial nanogrooves on the substrate owing to capillary action during a slow solvent evaporation process. The capillary behavior was readily manipulated by surface treatments over the glass spacers with self-assembled monolayers such as perfluorodecyltrichlorosilane (FDTS), *n*-decyltrichlorosilane (DTS), and 6-phenylhexyltrichlorosilane (PTS). As shown in Figure 10b, stronger capillary action was induced by PTS and piranha solution relative to DTS and FDTS. Consistently, the PTS treated spacers provided for a groove/ridge-like nanostructure on the bottom surface of resultant polymer films, highly aligned along the uniaxial nanogrooves on the substrate, while the FDTS treated spacers afforded a featureless structure. As a result, the resultant films obtained from a sandwich casting system comprising a PTS treated spacer showed significantly enhanced charge carrier mobilities by factors of 7.3 and 10.7 (from 2.9 to 21.3 and from 1.7 to 18.5 cm^2^·V^−1^·s^−1^) for poly[4-(4,4-dihexadecyl-4*H*-cyclopenta[1,2-b:5,4-b′]dithiophen-2-yl)-*alt*-[1,2,5]-thiadiazolo[3,4-c]pyridine] (PCDTPT) and poly[2,6-(4,4-*bis*-alkyl-4*H*-cyclopenta-[2,1-b;3,4-b0]-dithiophene)-*alt*-4,7-(2,1,3-enzothiadiazole)] (CDTBTZ), respectively (Figure 10c).

### 3.3. Centrifugal Force-Driven Film Deposition

**Figure 11:**
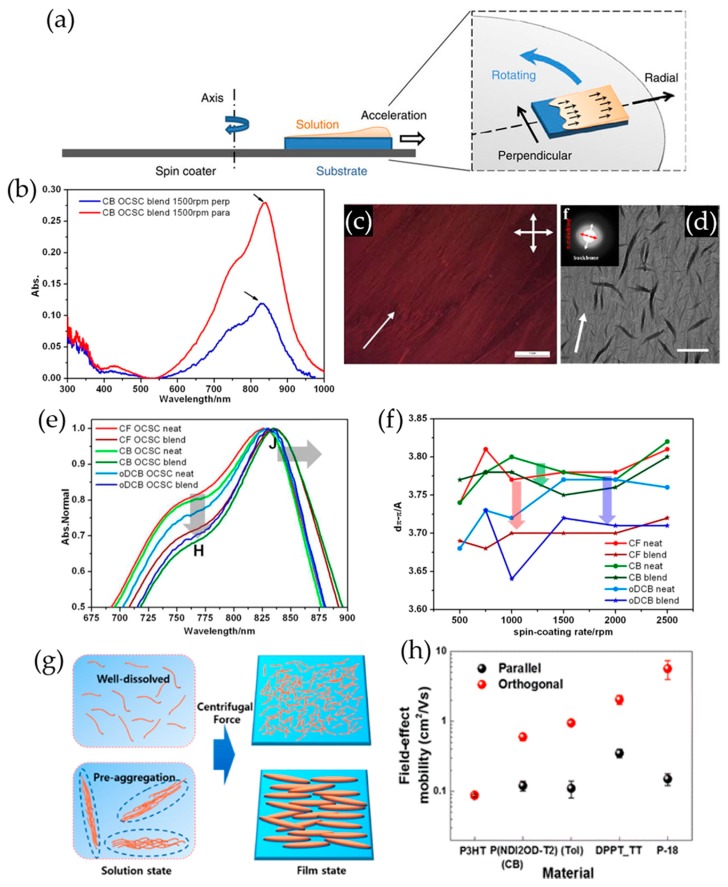
(**a**) Schematic illustration of the off-center spin coating process. Reproduced with permission from [96]. Copyright 2014 Nature Publishing Group. (**b**) Absorption spectra of PDBT-TT/PS blend films aligned parallel and perpendicular to the polarized direction. The arrow indicates the peak for calculating dichroic ratio. (**c**) Optical microscope image of a film prepared from a solution of PDBT-TT/PS blend by off-center spin coating in o-DCB under crossed polarizer and analyzer. The arrow indicates the radial direction during off-center spin coating. The scale bar represents 1 mm. The image was obtained at 45° with respect to the directions of crossed polarizer and analyzer. (**d**) TEM image of the corresponding film. The wide arrow indicates the radial direction during off-center spin coating, and the red and white arrows in electron diffraction patterns represent π–π stacking and backbone directions, respectively. The scale bar represents 1μm. (**e**) UV-vis-NIR absorption spectra for neat and blend films coated from solutions in different solvents. The direction of the arrows indicates an enhanced A_J_/A_H_ ratio. (**f**) Variation of π–π stacking distances with spin-coating rates for off-center spin coating neat and blend films coated from solution in different solvents. The direction of arrows represents the decrease of *d*_π–__π_ in blend films compared with neat ones. Reproduced with permission from [97]. Copyright 2015 American Chemical Society. (**g**) Schematic illustration of well-dissolved and pre-aggregated polymer in solution and solid-state. (**h**) Average mobilities of anisotropic P3HT, P(NDI2OD-T2), DPPT-TT, and P-18 OFETs. Reproduced with permission from [98]. Copyright 2015 American Chemical Society.

In addition to using capillary force, centrifugal force has also been demonstrated to be effective in achieving the alignment of conjugated polymer films [96,97,98,99]. An off-center spin-coating method was first reported by Yuan and coworkers, which induced the formation of a highly aligned, meta-stable crystal packing structure of 2,7-dioxtyl[1]benzothieno[3,2-b][1]benzothiophene (C8-BTBT) within a PS matrix. In this method, a substrate was placed away from the spin center. Then, a small amount of a C8-BTBT/PS blend solution was dropped on the substrate, followed by fast rotation of the spin-coater (Figure 11a). The resultant C8-BTBT/PS blend films exhibited highly improved hole mobility (from 3~16 to 25 cm^2^·V^−1^·s^−1^), attributed to the aligned crystalline grains combined with a slightly reduced in-plane intermolecular spacing. Recently, this strategy was applied to the conjugated polymer systems for achieving long-range alignment of polymer chains in resultant films. Wang et al. presented a unique optical anisotropy (i.e., dichroic ratio: ~2.75) of 3,6-bis(thiophen-2-yl)-*N*,*N*′-bis(2-octyl-1-dodecyl)-1,4-dioxo-pyrrolo-[3,4-c]pyrrole and thieno[3,2-b]thiophene (PDBT-TT)/PS blend films obtained via an off-center spin-coating approach (Figure 11b) [91]. It was revealed that such anisotropic feature resulted from the long-range alignment of PDBT-TT fibrillary bundles in the blend films (Figure 11c,d). In addition, both the intra- and interchain molecular order were enhanced, which was evidenced by the red-shifted max absorption, prevailing J-aggregation absorption, and smaller π–π stacking period (Figure 11e,f). Using an off-center spin coating, Kim et al. also directionally aligned various conjugated polymers such as P3HT, DPPT-TT, poly[(E)-1,2-(3,3′-dioctadecyl-2,2′-dithienyl)ethylene-*alt*-dithieno-(3,2-b:2′,3′-d)thiophene] (P18), and poly{[*N*,*N*′-bis(2-octyldodecyl)-1,4,5,8-naphthalenediimide-2,6-diyl]-*alt*-5,5′-(2,2′-bithiophene)} (P(NDI2OD-T2)) [98]. They asserted that pre-aggregated solutions are preferable to non-aggregated ones to form aligned morphologies of resultant films via off-center spin coating (Figure 11g). It was found that the alignment appears dominantly localized at the polymer film surface. Anisotropic charge transport was apparently observed from the aligned polymer films. Particularly, the P18 films aligned in parallel with the source-drain electrodes exhibited a charge carrier mobility (~7.25 cm^2^·V^−1^·s^−1^) 37-fold higher than that of the films vertically aligned to the source-drain (Figure 11h).

### 3.4. Mechanical Force-Assisted Film Alignment

**Figure 12:**
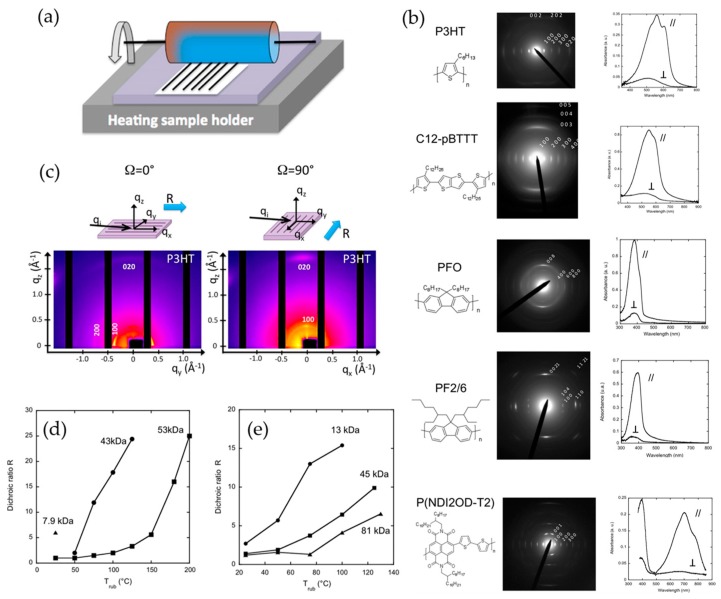
(**a**) Schematic illustration of the mechanical rubbing process. (**b**) Electron diffraction patterns and UV-vis absorption spectra (for parallel (//) and perpendicular (⊥) to the rubbing direction) of highly oriented various conjugated polymer films obtained by high temperature rubbing. The chemical structures of the corresponding polymers are shown on the left. (**c**) GIXD 2D maps obtained for rubbed P3HT films (43 k*D*_a_, *T*_rub_ = 180 °C). The 2D maps were recorded for the incident X-ray beam oriented parallel (Ω = 0°) and perpendicular (Ω = 90°) to the rubbing direction R as illustrated in the top sketches. Dichroic ratio at 605 nm forL (**d**) P3HTl and (**e**) C12-PBTTT with different molecular weight as a function of rubbing temperature. Reproduced with permission from [104]. Copyright 2014 American Chemical Society.

It is well known that mechanical rubbing is a typical technique to prepare an oriented polyimide film used for aligning liquid crystalline materials [94,95,96]. Interestingly, this process has also been employed to induce alignment of conjugated polymer films using a fabric or substrate at an elevated temperature [103,104,105]. Heil et al. showed that a spin-coated P3HT film can be aligned by mechanical rubbing with a piece of velvet at a temperature of 100 °C [103]. The dichroic ratio of the films was increased up to 5.1 upon mechanical rubbing, and no disorientation of the polymer chains was observed by a subsequent thermal annealing at 100 °C. As a result, the charge carrier mobility of OFETs was improved by up to 800% when the polymer films were rubbed perpendicular to the source-drain contacts. Recently, Biniek et al. demonstrated a mechanical rubbing technique that can be applied to a large palette of different π-conjugated systems such as p- and n-type semiconducting homopolymers and alternating copolymers (Figure 12b) [104]. In their procedure (Figure 12a), a polymer film placed on the translating sample holder was pressed by a cylinder covered with a microfiber cloth pressed under a pressure of 2 bar, followed by rotation of the cylinder at an elevated temperature. All the polymer films that were aligned by mechanical rubbing showed high dichroic ratios (Figure 12b). Particularly, a high dichroic ratio above 25 was observed from a RR-P3HT film rubbed at 180 °C. Further, they investigated the correlation between the degree of alignment and rubbing temperature and the molecular weight of the polymers. As shown in Figure 12c, the dichroic ratio increased with increased rubbing temperature while it decreased as the molecular weight increased.

Interestingly, another approach, which can afford aligned conjugated polymer films using mechanical compression, has been reported by Soeda et al. In a typical procedure, a relevant amount of poly[2,5-bis(3-hexadecylthiophene-2-yl)thieno(3,2-b)thiophene] (PB16TTT) solution was introduced dropwise onto the surface of an ionic liquid [105]. Then, it spread and covered the entire surface of the ionic liquid. Subsequently, the film was mechanically compressed in one direction by a glass blade and annealed to align the polymer main chains in a uniaxial direction on the surface of the ionic liquid. Finally, the resultant film that floated on the ionic liquid was transferred onto a substrate by horizontally attaching the substrate to the floating film. The corresponding film exhibited a dichroic ratio of 15.6, which is the highest record among directionally aligned PBTTT thin films. Commensurate with well-oriented its film morphology, a high charge carrier mobility exceeding 0.6 cm^2^ V·^−1^·s^−1^ was observed from aligned PB16TTT TFTs.

## 4. Conclusions

Controlling morphology of conjugated polymer thin films is as essential as synthesizing alternative polymer semiconductors that possess superior intrinsic electronic and mechanical properties for achieving high performance, large-area, flexible electronic devices suitable to a wide range of commercial applications. Typically, solidified conjugated polymer thin films exhibit lower intra- and intermolecular interactions and poor orientation of polymer chains, resulting in limited charge carrier mobility. Recently, huge progress in solution-based processing methods such as solution treatment and film deposition techniques, by which thin film morphology including crystallinity and orientation can be tuned, has been achieved. Solution treatments such as solvent solubility tuning using nonsolvents, addition of nucleation inducing agents, ultrasonication, UV irradiation, and their combinations tend to facilitate self-assembly of polymer semiconductors in solution state, resulting in highly ordered aggregates. The crystalline aggregates survive a film deposition process and in turn improve the crystallinity and thus charge transport characteristics of resultant polymer films, indicating no need for additional pre- and/or post-processing steps including polymer-dielectric interface treatment, solvent vapor annealing, and thermal annealing. Although the crystallinity of the films has been significantly improved by the solution treatments above, still the charge carrier mobility is limited due to poor grain orientations. As a response to this issue, alternative film deposition methods that can align polymer chains and their crystalline aggregates within the resultant films have been intensively developed in recent years. Particularly, numerous research efforts have been made toward the modification of the precedent techniques such as template-guided solution shearing and the development of alternative approaches using external forces such as capillary, gravitational, and mechanical force, owing to their strong potential to align polymer chains and aggregates. During solution processing, the formation and alignment of crystalline aggregates are critically influenced by a number of processing parameters such as solvent characteristics (e.g., solubility, volatility, polarity, viscosity, etc.), temperature, pressure, roughness and surface tension of substrates, fluid flow speeds, etc. Hence, a fundamental understanding of how processing parameters affect the morphological parameters including crystallinity, grain size, grain boundaries, and grain orientation, which are relevant to charge transport characteristics of resultant films, needs to be established in order to maximize device performance. In spite of significant advances in solution-based processing strategies, there are still many challenges to be surmounted, such as understanding the self-assembly mechanism, improving environmental stability, interface control between the polymer semiconductor and dielectric layer, and process scale-up. However, we believe that high performance organic electronics that possess low-cost, high environmental stability, and good mechanical properties will be realized in the near future by continuous worldwide research efforts.
